# A case series of two patients using closed-loop spinal cord stimulation: demonstration of the impact of real-time adjustments

**DOI:** 10.1093/jscr/rjag304

**Published:** 2026-04-28

**Authors:** Aviraj Soin, Sarah Kassis, Richard Joseph Witte, Ben Sloop, Cameron Brittain

**Affiliations:** The Ohio Pain Clinic, 7072 Corporate Way, Dayton, OH 45458, United States; Northeast Ohio Medical University, 4029 OH-44, Rootstown, OH 44272, United States; Wright State University, Colonel Glenn Hwy, Beavercreek, OH 45324, United States; Elite Pain Doctors, 6213 Snider Rd, Mason, OH 45040, United States; College of Medicine, Ohio University, 191 W Union St, Athens, OH 45701, United States

**Keywords:** spinal cord stimulation, closed-loop SCS, neural modulation, chronic back pain, sciatica

## Abstract

Closed-loop spinal cord stimulation (SCS) utilizes evoked compound action potential (ECAP) feedback to maintain constant neural recruitment. This case series reports outcomes from two patients treated with ECAP-controlled closed-loop SCS and evaluate changes in pain intensity and functional status. Two male patients presented with chronic lumbar radiculopathy and axial low back pain. At baseline, the patients reported severe pain with Numeric Rating Scale (NRS) scores of 7–8/10, accompanied by impaired sleep, standing tolerance, and walking capacity. Both patients underwent ECAP-controlled closed-loop SCS trials, during which real-time physiologic feedback was used to automatically adjust stimulation parameters and maintain consistent neural activation. Following treatment, both patients experienced substantial reductions in pain (NRS 1–2/10) along with marked improvements in sleep, standing, and walking tolerance. These improvements remained stable despite positional changes. ECAP-controlled closed-loop SCS was associated with significant improvements in functional outcomes while reducing the need for reprogramming and reintervention.

## Introduction

Chronic spinal pain, including low back pain (LBP) and neuropathic pain, remains a major clinical challenge with substantial impact on quality of life, sleep, mobility, psychological well-being, and socioeconomic productivity [1, 2]. LBP is often associated with overlapping but distinct conditions related to pain, such as referred pain and radiculopathy [3, 4]. Lumbar radiculopathy typically involves compression or irritation of the lumbar nerve roots and represents one of the most common neuropathic pain conditions, affecting ~40%–70% of individuals over their lifetime [5].

Axial LBP, in contrast, is a multifactorial syndrome involving both nociceptive and neuropathic mechanisms, with an estimated global prevalence of 458.8 million cases among the working-age population [6, 7]. Although lumbar radiculopathy and axial LBP differ in pathophysiology and clinical presentation, both conditions are often difficult to manage and may become refractory to conventional therapies [8, 9].

Conventional treatment strategies for lumbar radiculopathy include spinal manipulative therapy, function-specific physical training, and specific exercise [10]. Similarly, axial LBP is commonly managed with conservative management strategies such as education, therapeutic bracing, and exercise-based interventions [11]. However, a significant proportion of individuals fail to achieve sustained relief with these approaches. In such cases, spinal cord stimulation (SCS) has emerged as an effective treatment option, particularly when neuropathic pain features are prominent [12]. SCS is considered minimally invasive, reversible, and generally safe, with relatively low rates of complications and reintervention [13].

SCS involves the delivery of electrical impulses to targeted regions of the spinal cord, modulating the signaling pathways involved in pain, resulting in pain reduction and functional improvement [14]. Conventional open-loop SCS systems deliver stimulation at fixed parameters and are limited by their inability to compensate for physiologic variability, including postural changes and movement, which can alter neural recruitment over time [15]. To address these limitations, closed-loop SCS systems were developed.

Closed-loop SCS systems measure evoked compound action potentials (ECAPs) generated by dorsal column activation and automatically adjust stimulation output to maintain a predefined neural recruitment target [16]. This biologically responsive control mechanism enables stable stimulation delivery despite changes in posture, movement, or cerebrospinal fluid dynamics. These evolving insights provide important context for understanding the applicability of closed-loop neuromodulation across different pain syndromes. In this case series, we present two patients with distinct pain phenotypes, lumbar radiculopathy and axial LBP, treated with ECAP-controlled closed-loop SCS, highlighting both clinical outcomes and mechanistic considerations.

## Case-series presentation

### Case 1

**Figure 1:**
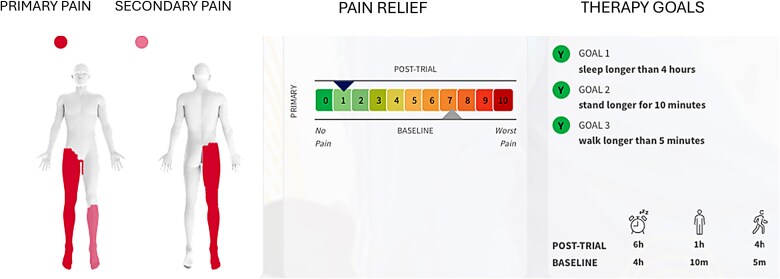
Patient with lumbar radiculopathy before and after the closed-loop spinal cord stimulation.

The first male patient, over the age of 50, presented with lumbar radiculopathy, with pain duration > 6 months and pain that was refractory to pharmacotherapy and physiotherapy. Baseline pain severity was assessed using the Numeric Rating Scale (NRS) [17], with scores of ~7/10. The primary complaint was of right lower extremity pain that radiated into his foot, with secondary pain involving the left lower extremity from the knee to the foot. At baseline, sleep duration was limited to ~4 hours, standing tolerance to 10 minutes, and walking tolerance to 5 minutes (Fig. 1).

**Figure 2:**
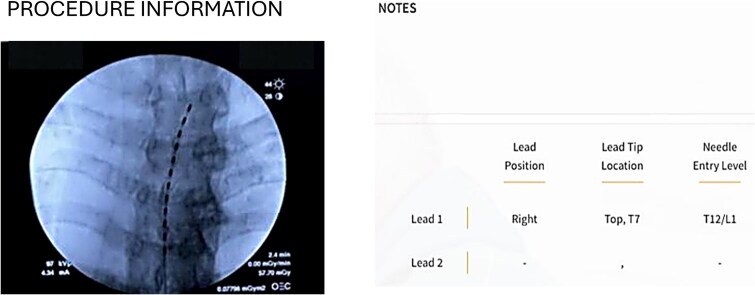
Fluoroscopic imaging of the spinal cord stimulator demonstrated a lead spanning the T7 vertebral body.

The patient underwent a trial of ECAP-controlled closed-loop SCS. A single lead was placed spanning the T7 vertebral body under fluoroscopic guidance (Fig. 2).

**Figure 3:**
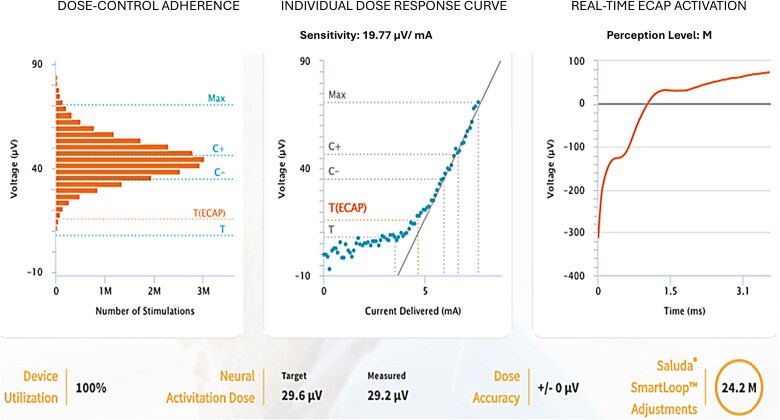
Closed-loop stimulation performance demonstrated highly stable neural activation in a patient with lumbar radiculopathy.

During the one-week trial, ~24.2 million automated closed-loop adjustments were performed. Dose accuracy remained within ±0 μV, with a target ECAP of 29.6 μV and a measured ECAP of 29.2 μV. Device utilization during the trial was 100%. The most frequently used stimulation program included a pulse width of 250 μs and a frequency of ~40 Hz (Fig. 3).

By the second day of treatment, the patient reported substantial clinical improvement, describing himself as feeling “like a new man.” Sleep duration increased to ~6 hours, standing tolerance improved to 1 hour, and walking tolerance increased to 4 hours without pain. Pain intensity decreased to an NRS score of 1/10 (Fig. 1). The patient also reported the ability to drive without discomfort, compared with a pretreatment driving tolerance of ~15 minutes due to severe pain. Following a successful trial, permanent implantation was performed with lead placements across the T7 vertebral body under the guidance of fluoroscopy (Fig. 3).

### Case 2

**Figure 4:**
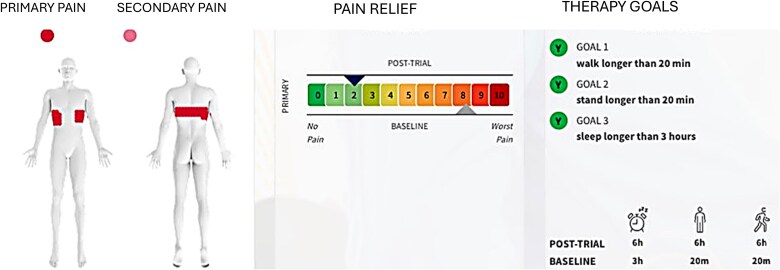
Patient with axial low back pain before and after the closed-loop spinal cord stimulation.

The second male patient, over the age of 50, reported axial LBP characterized by localized pain in the lower back and anterior abdominal region. At baseline, the patient reported an NRS pain score of 8/10, with sleep limited to ~3 hours per night and standing and walking tolerance restricted to 20 minutes (Fig. 4).

**Figure 5:**
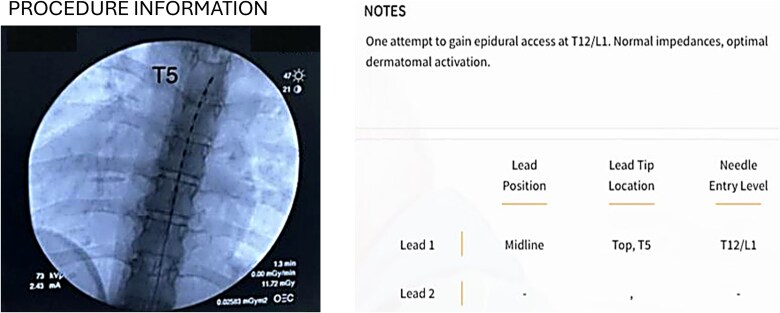
Fluoroscopic imaging of the spinal cord stimulator demonstrated a lead spanning T5–8 vertebral bodies.

The patient underwent a closed-loop SCS trial. Epidural access was obtained at T12/L1, and the lead was positioned spanning the T5–T8 vertebral levels under fluoroscopic guidance (Fig. 5).

**Figure 6:**
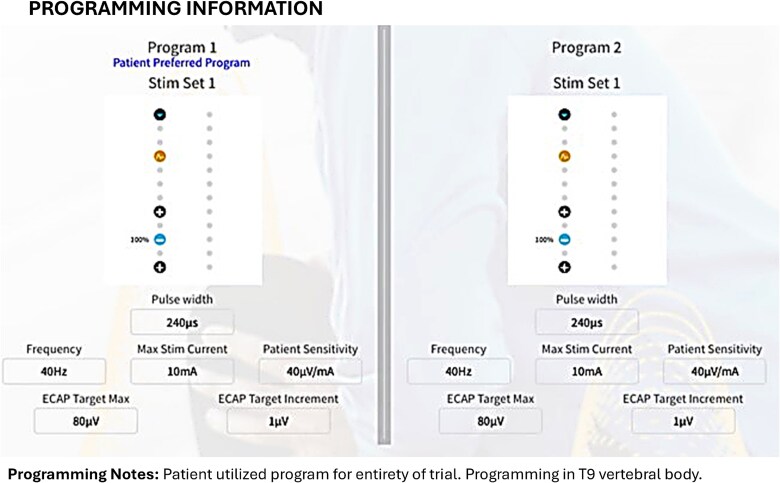
Programming information utilized by a patient with axial low back pain for his entire treatment period.

Closed-loop programming was performed using parameters detailed in Fig. 6. During the one-week trial, ~24.0 million automated adjustments were recorded, with dose accuracy maintained within ±5 μV. The target ECAP was 19.4 μV, and the measured ECAP was 16.4 μV. Device utilization was 100% throughout the trial period. Stimulation parameters included a pulse width of ~250 μs and a frequency of 40 Hz.

Following treatment, the patient demonstrated marked improvement across all the predefined therapy goals. Sleep duration increased to ~6 hours, standing and walking tolerance improved to ~6 hours, and pain intensity decreased to an NRS score of 2/10 (Fig. 4). The patient was discharged with sustained symptomatic improvement.

## Discussion

Both patients in this case series experienced substantial reductions in pain intensity, with NRS scores decreasing from 7–8/10 to 1–2/10, accompanied by meaningful improvements in sleep, standing, and walking capacity. These outcomes highlight the potential clinical utility of ECAP-controlled closed-loop SCS across distinct spinal pain phenotypes, including lumbar radiculopathy and axial LBP.

Our findings align with prior reports demonstrating the effectiveness of a closed-loop-SCS case study involving metastatic colon cancer–related lumbar radiculopathy, which reported significant pain reduction and functional improvement following SCS treatment [18]. A prospective multicenter study observed sustained improvements in quality of life and sleep over 24 months, with 82.8% of the patients reducing opioid use [19]. Similarly, a multicenter prospective study of ECAP-controlled closed-loop SCS in nonsurgical chronic back pain reported that 79% of the patients achieved ≥50% pain reduction at 12 months, with 48% achieving ≥80% pain relief [20]. Network meta-analyses have further demonstrated the superior efficacy of SCS approaches compared with conventional medical management strategies [21]. Similarly, a network meta-analysis was performed to compare the outcomes of SCS with conventional medical management strategies. SCS approaches (conventional and novel) proved their superior efficacy over conventional medical management strategies [conventional SCS: 3 odds ratio (OR), 95% confidence interval (CI), 1.49–6.72; novel SCS: 8.76 OR, 95% CI, 3.84–22.31] for pain intensity [21].

Although these studies were in alignment with our findings, they differed primarily in the clinical context, as we included two patients with different pain types, one with lumbar radiculopathy and the other patient with axial LBP. We also demonstrated a clear and significant reduction in pain intensity and medication usage, as after treatment our patients were discharged with improved pain scores and functional outcomes. Other studies also reported improvement in the quality of life, sleep, and function during 24 months of follow-up, and 35%–82.8% of patients reduced their opioid intake [19, 22]. Overall, prior studies offer long-term and population-based validation of closed-loop SCS, and our case series may complement them by providing individual cases with different types of pain and clinical outcomes.

No doubt SCS proved to be an effective treatment modality for pain reduction and improved functional outcomes. However, there is a need to understand the mechanism involved during SCS treatment. Based on the “gate control theory for pain transmission” spinal and supraspinal mechanisms are involved [23]. In addition, SCS also increases the synthesis and expression of encephalin and dynorphin within the dorsal horn of the spinal cord, which is also helpful in the release of substance P and serotonin [24, 25]. Furthermore, it is also helpful in modulating adrenergic and cholinergic neurotransmission by releasing noradrenaline and acetylcholine in the dorsal horn of the spinal cord [26, 27].

Beyond analgesia, closed-loop SCS may reduce reliance on opioid medications and improve long-term functional outcomes. Although the initial cost of closed-loop systems is higher, improved success rates and the reduced need for adjunctive therapies may yield favorable cost-effectiveness over time. Further research is needed to directly compare economic outcomes with traditional management strategies.

## Conclusion

ECAP-controlled closed-loop SCS demonstrated significant and stable improvements in pain and functional outcomes in two patients with distinct chronic spinal pain conditions. Real-time ECAP-guided modulation allowed continuous adjustment of stimulation, facilitating consistent neural recruitment. These findings support closed-loop SCS as a promising advancement in personalized pain management and warrant further population-based evaluation.
